# Applying Answer Set Programming for Knowledge-Based Link Prediction on Social Interaction Networks

**DOI:** 10.3389/fdata.2019.00015

**Published:** 2019-06-26

**Authors:** Çiçek Güven, Martin Atzmueller

**Affiliations:** Computational Sensemaking Lab, Department of Cognitive Science and Artificial Intelligence, Tilburg University, Tilburg, Netherlands

**Keywords:** modeling social media, social network analysis, link prediction, answer set programming, knowledge-based

## Abstract

Link prediction targets the prediction of possible future links in a social network, i. e., we aim to predict the next most likely links of the network given the current state. However, predicting the future solely based on (scarce) historic data is often challenging. In this paper, we investigate, if we can make use of additional (domain) knowledge to tackle this problem. For this purpose, we apply answer set programming (ASP) for formalizing the domain knowledge for social network (and graph) analysis. In particular, we investigate link prediction via ASP based on node proximity and its enhancement with background knowledge, in order to test intuitions that common features, e. g., a common educational background of students, imply common interests. In addition, then the applied ASP formalism enables explanation-aware prediction approaches.

## 1. Introduction

Social interaction networks are mediated via social media in various forms and can be modeled using many diverse approaches, particularly using network theory. According to the idea of social interaction networks (Atzmueller, 2014), we adopt an intuitive definition of social media, regarding it as online systems and services in the ubiquitous web, which create and provide social data generated by human interaction and communication (Atzmueller, 2012). Specifically, we target link prediction for predicting future links in a network using background knowledge, formalized by logical formalisms. These allow to provide crucial domain knowledge: in scenarios when historic (link) data is still scarce—similar to the cold-start problem for link prediction— domain knowledge can complement structure-based link prediction. Thus, we utilize domain knowledge to enrich interaction networks, leading to knowledge-based feature-rich networks.

In this paper, we propose to use Answer Set Programming (ASP) for formalizing domain knowledge in order to enable hybrid link prediction (an approach that combines using the network itself as well as background knowledge to predict future links) in a social interaction network. ASP is a form of declarative programming that is used for difficult (NP hard) search problems, c. f., Lifschitz (2008). Here, ASP is relevant since it allows to specify interesting structures and patterns in a compact way, and due to its strength in including background knowledge by facts (and rules) intuitively. The ASP approach involves passing the (graph) structure and the conditions, and returns the (answer) set satisfying the conditions.

**Figure 1:**
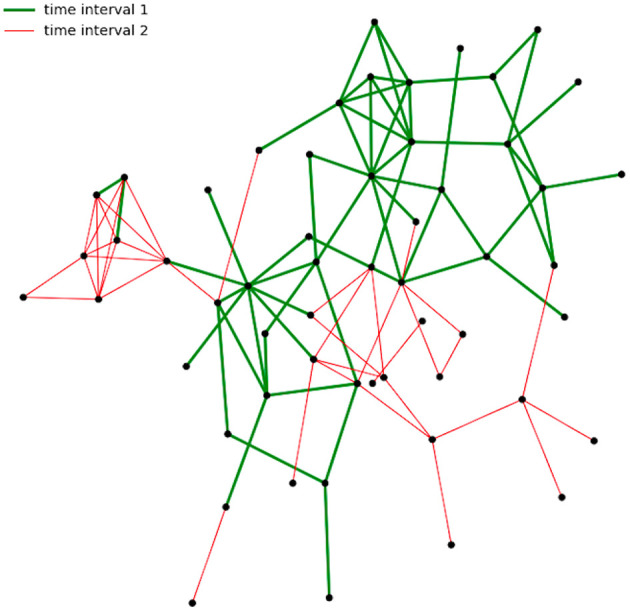
Interaction network (links are split into subsets based on time).

The proposed approach is exemplified using a real-world data set capturing networks of face-to-face proximity at a student event. In the interaction network, which is studied for the link prediction task, there are actors (nodes) who only start interacting with the other actors after a while. In network terms, that means they are disconnected from the rest of the nodes given that a connection is there when there is an interaction. This is known in the literature as the cold start problem, (Leroy et al., 2010). An illustration of this is shown in Figure 1; links are split into two classes based on time. The links which correspond to interactions in the earliest interval, namely ‘time interval 1’ have color green, and are the thicker ones, whereas the color of the edges for the second interval is red.

In this example, we observe that there are nodes which only have connections with red colored edges; this means, that the corresponding interaction happened after the first interval. For those, we cannot apply, e. g., neighborhood features or path-based features for prediction, since no prior links/paths exist between these nodes and the others in the first interval. However, this data is complemented by attributive nodal information, which will be formalized as domain knowledge. Then, these might be informative to make predictions. That is, links between actors can be predicted based on a relation between actors and attributive information. With ASP, it is easy to incorporate such domain knowledge in the form of simple logical predicates and rules. That is why we consider it as an ideal tool in order to incorporate additional information.

It is important to note, that the purpose of this paper is not on analyzing specific patterns and insights on link prediction in social interaction networks, or to show that an ASP approach results in the best performance. Instead, we aim to provide a “proof of concept” of its applicability for link prediction, and to demonstrate its advantages like explainability and enabling a simple formalization and refinement of domain knowledge. The contribution of this paper is thus 2-fold:

1. We introduce the application of ASP as a novel approach for link prediction.
2. We demonstrate how to improve link prediction with contextual domain knowledge modeled using ASP.

The rest of the paper is structured as follows: section 2 discussed necessary background including basic definitions on graphs, and a brief introduction into ASP. After that, section 3 discusses related work. Next, section 4 outlines the proposed method using ASP for link prediction. Then, section 5 presents our results. Finally, section 6 concludes with a summary and outlines interesting directions for future work.

## 2. Background

In this section, we define basic concepts in graph theory that are relevant for this paper. For further background in graph theory we refer the work of Diestel (2017). Next, we provide a brief overview on ASP.

### 2.1. Basic Definitions: Graph Theory and Link Prediction

A graph *G* is an ordered pair (*V, E*) consisting of a set of vertices (nodes) and a set of edges. An edge (*u, v*) consists of a pair of nodes *u, v* representing a relationship between them. A social network can be abstracted by a graph, where actors correspond to nodes and the links in between them corresponds to edges. A node *v* is a neighbor of (adjacent to) a node *u* if there is an edge (*u, v*) between them. Γ(*u*) stands for the set of neighbors of a node *u*. Let *G* = {*G*_*t* = *o*_, *G*_*t* = 1_, ⋯ , *G*_*t* = *n*_} be a temporal sequence of evolving graphs where *G*_*t* = *i*_ = (*V*_*t* = *i*_, *E*_*t* = *i*_). For link prediction on such sequences, given *t* = *n* the goal is to predict the structure of a graph in *t* = *n*+1, i. e., *G*_*t* = *n*+1_. Specifically, we try to identify pairs (*u, v*), such that *u, v* ∈ *V*_*t* = *n*+1_ and (*u, v*) ∈ *E*_*t* = *n*+1_.

Prominent approaches for link prediction consider similarity scores between pairs of nodes, e. g., based on neighborhoods of pairs of nodes. Here, we will enhance link prediction based on neighborhood-based similarity scores with background knowledge. As one prominent neighborhood-based similarity score, we use the *Common neighbors* score: It counts the number of common neighbors of a pair of nodes. Given, (*u, v*) the pair of nodes under observation, the common neighbors can formally be written as:

CN(u,v)=|Γ(u)∩Γ(v)|

### 2.2. Overview on Answer Set Programming

Answer Set Programming (ASP) (Niemelä, 1999) is a declarative problem solving approach; it is one of the three major logic programming families next to Prolog and Datalog. Logic programming is a programming paradigm mainly based on formal logic; such a program consists of facts and rules about the problem domain expressed as sentences in logical form. Given a problem, ASP aims to find one or several possible solutions; these are the so-called answer sets, i. e., all possible sets of facts that are consistent with the facts stated earlier) to the original problem (c. f., e. g., Gebser and Schaub, 2016; Kaufmann et al., 2016). This requires expressing the problem in a formal way. So, we transform and model the problem in the form of a logic program, which consists of rules and variables. A special program, i. e., the *grounder* then eliminates all instances of the variables and replaces them by ground terms (which can be considered as “values,” i. e., propositional atoms) in the language. This facilitates the application of the subsequent step, i. e., applying the answer set *solver*, which typically works on variable-free programs. Finally, the resulting propositional program, which is free of variables, only consists of propositional atoms. This is then the input to the solver which computes the answer sets. Those are all possible sets of facts that are consistent with the facts stated earlier to the original problem. For a more detailed discussion, we refer to e. g., (Niemelä, 1999; Gebser and Schaub, 2016; Kaufmann et al., 2016).

The ASP rules include user defined predicates and variables, as in the following example for common neighbors (*CN*):


  
  CN(X, Y, Z) :-edge(X, Y), edge(X, Z),
  not edge(Y, Z), Y!=Z. 
 


In this notation, “,” means “and,” “:-” means “if,” and “not” stands for negation. Here, “CN ,” and “edge ” are examples of user defined predicates, which can be true or false for object(s) represented by a specific term replacing a user defined variable(s) such as ‘(1,2) '. The rules without any conditions are called facts. Our example rule is used to formalize the following information: *X* is a common neighbor of a pair of distinct vertices *Y* and *Z*, if there are edges between pairs *X, Y* and *X, Z* but not between *Y* and *Z*. The if symbol ‘:- ’ is omitted for the facts, so that ‘edge(1,2). ’ is a fact.

The solution to a problem is called an “answer set", which consists of propositions that are supposed to be true in the answer set. A solution to the above rule and the two facts ‘edge(1,2). ’, and ‘edge(1,4). ’ is the answer set containing these facts and the propositions ‘CN(1,2,4). ’, and ‘CN(1,4,2). ’.

We used ASP to enhance link prediction in a network with background knowledge and used a small data set for this proof of concept. However, ASP is designed for NP-hard problems as stated earlier and finds its applications in large instances of industrial problems, since it offers a rich representation language and high performance solvers; some recent applications are listed in Falkner et al. (2018). Some examples of ASP solvers that are considered to be efficient are Smodels (Syrjänen and Niemelä, 2001), WASP (Dodaro, 2013), Clasp (Gebser et al., 2012) and Clingo (Gebser et al., 2014b). Clingo^1^ itself combines a powerful grounder (Gringo) with Clasp (for solving) into an integrated system. For ease of use, and due to its efficiency (e. g., Guyet et al., 2018; Schäpers et al., 2018), we utilized Clingo in the context of this paper.

## 3. Related Work

The focus of link prediction is the dynamics and mechanisms in the creation of links between the parties in social networks (Liben-Nowell and Kleinberg, 2003). The purpose is to learn a model for predicting the links accurately. There is already a large body of research for link prediction concerning *online* social networks, e. g., (Katz, 1953; Adamic and Adar, 2003; Liben-Nowell and Kleinberg, 2003; Murata and Moriyasu, 2007; Lü and Zhou, 2010; Scholz et al., 2013, 2014) considering neighborhood-based and path-based measures. A first comprehensive fundamental analysis was done by Liben-Nowell and Kleinberg (2003), where the link prediction problem was defined as the search to carefully predict edges that will be added to a given snapshot of a social network during a given interval, using network proximity measures. This shows a strong connection to the approach to this paper, while we apply a novel approach, i. e., ASP for performing the search. In addition, we also include domain knowledge for a knowledge-based link prediction approach, also tackling the common cold start problem in link prediction (Leroy et al., 2010).

Link predictions can be used for different prominent applications: recommending and suggesting promising interactions between two individuals in such a social network (Li and Chen, 2009; Papadimitriou et al., 2011), the prediction of missing links, (Liben-Nowell and Kleinberg, 2003), and improving collaborative filtering (Huang et al., 2005). In this paper, we mainly focus on the perspective of utilizing link prediction for recommendation and collaborative filtering, while also target explainability and transparency of the predictions which is also facilitated by our proposed approach.

To the best of the authors' knowledge, the idea of merging Answer Set Programming and link prediction in the context of social networks is new. De Raedt et al. (2007) studied a probabilistic version of Prolog, to discover links in large network of biological concepts. The probabilistic Prolog would then aim to compute the success probability for the existence of a link between nodes such as genes and diseases. Furthermore, there have been earlier studies relating ASP and social network analysis: Jost et al. (2012) modeled a way to suggest new interactions related to events in a social network for a personal assistant of the network platform (EasyReach) which monitors interactions. A study relating social networks with ASP in the privacy and security context is described in Hu et al. (2013). There, multiparty access control for online social networks is studied. Marra et al. (2014, 2016) studied properties of social networks, and information diffusion in Social Network Analysis. They applied ASP for analyzing properties of social networks, in a multi-social-network setting. The study of Seo et al. (2013) also combines social network analysis and logic programming. In that study a high-level graph query language SociaLite based on Datalog is proposed, due to its expressive power and efficiency, an tested on real life social graphs. We have a similar motivation in terms of the ease of use, and of expressivity, where we target explicative link prediction in the context of social networks, utilizing topological network information as well as attributive relations. Furthermore, explainable social network analysis is a further feature of the ASP-based approach, where first approaches in the context of explicative data mining (Atzmueller, 2017, 2018) have been discussed by Masiala and Atzmueller (2018a,b).

## 4. Methods

In the following, we outline our method for link prediction using ASP. The main strength of ASP is its intuitive way to state a problem, also allowing to scale the problem up easily, and the availability of computationally powerful ASP solvers. For this study, the former two points are more relevant since in our application context we utilize a relatively small data set so far. As an ASP solver, we use Clingo (Gebser et al., 2014a) embedded in Python.

Below, we will first illustrate our approach via a small hypothetical example. Then we will describe the data set, and finally we will discuss our findings on the data set.

### 4.1. Example

**Figure 2:**
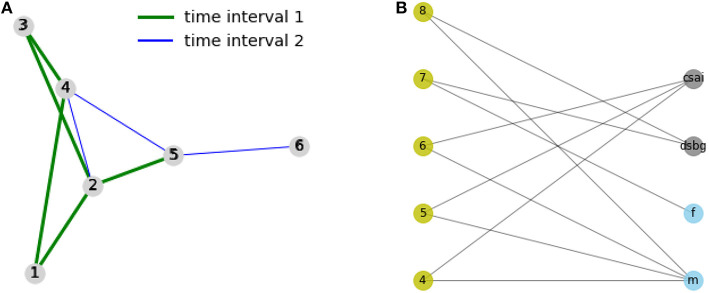
Example: interaction and attributive/knowledge-based networks. **(A)** Interaction network (links are split into subsets based on two different time intervals. **(B)** Graph capturing attributive data: students on the left, and attributive information on the right.

We consider a social interaction network between students as actors, and attributive information collecting information such as gender, affiliation, and area of study of the students. For those, we provide two according network structures: one indicating the interactions, the other (bimodal) one modeling information of the students as actors in the network. Regarding the left network, the graph *G* shown in Figure 2 represents interaction between actors at an event, split into two time frames. The edges *E*_1_ = {(1, 2), (2, 3), (1, 4), (3, 4), (2, 5)} represent the interactions in the first interval *T*_1_, *E*_2_ = {(2, 4), (1, 5), (3, 5), (2, 6)} represent the interactions that happened in the second time interval *T*_2_ afterwards. The bipartite graph *G*_*A*_ shown on the right of Figure 2 represents the choices of the attributive information provided as background knowledge. The nodes 4, 5, 6, 7, 8 represents students, and the nodes *f, m* represent their gender (*f*: female, *m*: male). The nodes *dsbg, csai* are standing for the master programs the students are enrolled to, e. g., “Cognitive Science and Artificial Intelligence" or “Data Science for Business and Governance". The edges in *E*_2_ are aimed to be predicted by using information coming from prior interactions captured by *E*_1_ as well as captured by background knowledge given *G*_*A*_.

The following code predicts a link between a pair of nodes in *G* for *T*_2_ if they have two common neighbors in *G* during *T*_1_ or *G*_*A*_. That is, a link is predicted for a node pair *u, v* without an existing link in the interaction graph for *T*_1_ [(*u, v*) ∉ *E*_1_] when they are similar in terms of their neighbors, or when they are similar based on their respective attribute values, in this case having the same gender and following the same program are necessary. Then the code compares the links in *G* for *T*_2_, *E*_2_ (which we can see as a test set), and returns the matches between the predicted links *E*_2*pred*_ and the test set. The ASP program is composed of two parts: The *facts* describing the networks, and the *rules* for inferring the prediction.


  #**const** n=2.
  #**const** n_attrib=2.
  
  % ASP facts
  % Defining the networks/graphs
  node(1.6). % Nodes of the interaction graph
  edge(1, 2).  edge(1, 4). edge(2, 5). edge(2, 3). edge(3, 4). % Edges, first time interval
  test(2, 4). test(4, 5). test(5, 6). % Edges, second time interval (test set)
  % Nodes and edges of the attributive graph:
  node_attrib(4.8). node_attrib(csai). node_attrib(dsbg). node_attrib(f). node_attrib(m).
  edge_attrib(5, csai). edge_attrib(8, dsbg). edge_attrib(7, dsbg).
  edge_attrib(4, csai). edge_attrib(6, csai). edge_attrib(5, m). edge_attrib(4, m).
  edge_attrib(8, m). edge_attrib(7, f). edge_attrib(6, m).
  
  % ASP rules
  % This is an undirected graph,  hence there is symmetry in edges.
  edge(Y, X) :- edge(X, Y).
  edge_attrib(Y, X) :- edge_attrib(X, Y).
  % X is a common neighbor of Y and Z where they are not connected.
  c(X, Y, Z) :- edge(X, Y), edge(X, Z),  not edge(Y, Z), Y!=Z.
  c_attrib(X, Y, Z) :- edge_attrib(X, Y), edge_attrib(X, Z),  not edge_attrib(Y, Z), Y!=Z.
  % a link is predicted when there are 2 common neighbors in the interaction graph
  cn_lp(Y, Z) :- node(Y), node(Z), not edge(Y, Z), Y!=Z,  n=#count{X:c(X, Y, Z)}.
  % a link is predicted  when there are 2 common neighbors in the attributive graph
  cn_lp(Y, Z) :- node(Y), node(Z), not edge(Y, Z), Y!=Z,  n_attrib=#count{X:c_attrib(X, Y, Z)}.
  test(Y, X) :- test(X, Y).
  % The match rule compares the predicted set of links with the test set
  match(X, Y) :- test(X, Y),  cn_lp(X, Y).
  
  #show cn_lp/2.
  #show match/2.
 


This example is designed in such a way that, there is 100% overlap between the predicted links and the test set. Thus, the output is:


 
  match(2,4) match(4,5) match(5,6) match(6,5) match(5,4) match(4,2) cn_lp
            (4,2) cn_lp(2,4) cn_lp(1,3) cn_lp(3,1) cn_lp(4,6) cn_lp(5,6) cn_lp
            (6,4) cn_lp(5,4) cn_lp(6,5) cn_lp(4,5)
 


It is easy to see that–depending on the formalization of the predicates and rules used in the ASP program, the answer set itself can accommodate helpful explanations of why a link was predicted. This can be supported by a trace of the applied rule structure, e. g., utilizing a reconstructive explanation methodology (Wick and Thompson, 1992; Atzmueller and Roth-Berghofer, 2010), complemented by further background knowledge and/or context information from the network structure.

Since the graph derived from the attributive information connects the students to other parameters, a prediction based on its common neighbors will predict links between students when constructed as above. The rules can be modified in such a way that for a constant *n*, where Γ_*A*_(*x*) stands for the neighborhood of node *x* in *G*_*A*_, *E*_2*pred*_ stands for the predicted edges for *T*_2_:

∀*u, v, x, y* ∈ *V* ∣ (*u, v*) ∉ *E*_1_, |Γ_*A*_(*u*) ∩ Γ_*A*_(*v*)| = *n* ⇒ ∀*x* ∈ Γ_*G*_1__(*u*)\Γ_*G*_1__(*v*), (*x, v*) ∈ *E*_2*pred*_ and ∀*y* ∈ Γ_*G*_1__(*v*)\Γ_*G*_1__(*u*), (*x, u*) ∈ *E*_2*pred*_.

### 4.2. Data Set Description

For this study, we utilized a real life data set, which had been collected during a student event. This included information on face-to-face interactions and attributive information including gender, academic degree, age group, area of studies.^2^ For that, active proximity tags based on Radio Frequency Identification technology (RFID-chips) developed by the SocioPatterns Collaboration^3^ were applied. These are able to detect face-to-face interactions at large scale, using the radio packets exchange between two devices provided that the devices are in a distance of 1–1.5 m, and the parties remained in contact for at least 20 s. An interaction ends, when no packets are detected within a 20 s interval. The sensor data is used to construct social interaction networks capturing offline interactions between people. For more details on the data preprocessing, we refer to Barrat et al. (2010).

For constructing feature-rich networks, we utilized the data set focussing on its two components: One is capturing the interactions collected via sensors between students, and the other one is based on the given attributive information. The interaction data set contains data from 56 students attending the student event. First, using the proximity contacts, we generated a social interaction network. Then, an edge {*u, v*} is created, if a face-to-face contact with a duration of at least 20 s among participants *u* and *v* was detected. There were 340 interactions with the lower bound of 20 s, the maximal interaction length being 1,042 s (on average 69.5 s), over the course of 8 hours. After removing duplicate edges (only the first interactions are kept between parties in case there were more than one interaction), only 97 edges are left. These edges are split into two subsets *E*_1_, and *E*_2_ with corresponding graphs *G*_1_, *G*_2_ while the order based on time is preserved with ratio (6:4).

The attributive data set is relevant to capture the similarities based on the attribute values, which is structured as a bipartite graph *G*_*A*_. One of the partitions consists of the student ids (anonymous) and the other partition consists of attributes about gender, age group, academic degree, area of studies. For instance, there is a node corresponding to value ‘female’ for the gender attribute, “Data Science” for the area of studies. There is an edge between the node representing a student and the nodes representing the attribute. This resulted in a data set consisting of two columns corresponding to the sets of nodes representing the partition, where each row represents an edge. There are 456 rows in this data set, and 124 vertices partitioned into two sets as described above for students and attributive information of respective sizes 76 and 48. Some characteristics of the graphs *G*_*A*_, *G*_1_, *G*_2_ can be seen in Table 1. The sparsity in the interaction graphs makes link prediction a hard problem there.

**Table 1:** Network characteristics: Attributive network, and the interaction network in two time intervals.

**Characteristics**	**G_*A*_**	**G_1_**	**G_2_**
Number of nodes	124	47	40
Number of edges	456	59	38
Density	6%	5.5%	4.9%

## 5. Results and Discussion

We first focused on the cold start problem. There are 9 nodes which showed up in the second time interval. There are 14 edges for these nodes in *E*_2_. For any pair of vertices in the graph, if there is an edge between them in the test set, then that is an actual positive, otherwise actual negative. A match between the predicted and actual positive is a true positive. We predicted edges for the newcomers based on a simple similarity measure in *G*_*A*_. We predicted an edge between a pair of students if there had been no edge between them in *G*_1_, and they had *n* common neighbors in *G*_*A*_ graph where *n* is in {4, 5}. This implied 7 true positives, and 65 predicted positives out of 315 possible edges in *G*_2_. These imply a precision of 10.7%, a recall of 50% and an F1 score of 18%.

The following rules are used to augment the common neighbor method described by an example above, with the formalized background knowledge coming from the attributive information. An edge is predicted between a pair of vertices in *V*_2_, if there is no such edge in *G*_1_, these vertices are distinct and they have four or five common neighbors in *G*_*A*_.


 
  #**const** n_attrib1=4.
  #**const** n_attrib2=5.
  attributive_edge(Y,X):- attributive_edge(X,Y).
  c_attrib(X,Y,Z) :- attributive_edge(X,Y), attributive_edge(X,Z), not 
            attributive_edge(Y,Z),Y!=Z.
  pn(Y,Z) :- e_2_node(Y), e_2_node(Z), Y!=Z, not e_1_edge(Y,Z), n_attrib1
              =#count{X:c_attrib(X,Y,Z)}.
  pn(Y,Z) :- e_2_node(Y), e_2_node(Z), Y!=Z, not e_1_edge(Y,Z), n_attrib2
              =#count{X:c_attrib(X,Y,Z)}.
 


We chose the number of common neighbors as the similarity metric, since it is s standard metric, and it is also very explainable and interpretable, as also discussed above. Using ASP we first predicted links based on common neighbors only–utilizing the interaction network. We predicted a link between a pair of non-adjacent nodes in *G*_1_, when they have *n* common neighbors, for different values of *n*, and compared these with *G*_2_ = (*E*_2_, *V*_2_), treated as the ground truth for this problem. Given that the network *G* has low density all edges considered (i. e., the data is not balanced across classes) accuracy is not a good metric, hence we look into precision recall and F1 score only, see Table 2. There are 38 edges in *G*_2_, which is the size of actual positives, $\left(\begin{array}{c} 40\\ 2 \end{array}\right)$ = 780 possible edges, and 742 actual negatives, that is the difference between possible and existing edges.

**Table 2:** Link prediction evaluation metrics.

**Number of common neighbors**	**Graph used for prediction**	**True positives**	**Predicted positives**	**Precision**	**Recall**	**F_1_**
≤ 4	*G*_1_	6	31	19.4%	7.7%	11.0%
≤ 4	*G*_1_	16	170	9.4%	42.1%	15.4%
∈ {4, 5}	*G*_*A*_					

We see in Table 2, link prediction solely on interaction data does not work well with the common neighbors metric: We only achieve an F1 score of 11.0%. We noted earlier, one limitation of using interaction data is the cold start problem. Here *V*_1_\*V*_2_ = 16, *V*_1_\*V*_1_ = 9. That is a big community change, 16 people left and 12 new people arrived. That is a potential explanation to the performance. However, even if we neglect the cold-starters, focusing on the intersection of nodes in *G*_1_ and *G*_2_ then we still obtain rather comparable bad results, which we also verified using the linkpred package^4^ using the standard common neighbors, preferential attachment and rooted pagerank metrics. When we start adding new information based on the attributive information in *G*_*A*_, the number of true positives starts increasing as well. In our results, we see an increase on the cold-starters of 18%, leading to an overall F1 measure of 15.4% which clearly outperforms the baseline. A refined exploitation of the background knowledge can then lead to further improved evaluation metrics, e. g., by including social theories and extending our applied simple common neighbors strategy.

Link prediction is quite difficult for this data set, due to sparsity and the cold start problem. Given the results, we can argue common neighbors is not a very strong predictor for future links for this data set. With the attributive information data we see an increase in false positives (wrongly predicted links) decreasing the precision, and F1 but since correctly predicted links also increased, recall increases slightly. It is important to note that we so far applied only a simple strategy for formalizing background knowledge: The purpose here is to propose an approach to the link prediction problem, not to find the best performing method. We aim to refine the model using the attributive information by formalizing appropriate background knowledge, in order to explore options for improving link prediction in future work.

We treated any attribute value equally here, where as in reality, some attributes will be more informative than others. Also, more common attribute values might be less informative. The results can then be improved by exploring those. Overall, ASP remains an ideal way to incorporate and test that additional background knowledge with its flexibility. For example, ASP can be used to incorporate further insights about the population studied by looking further into background data. Some observations whose impact into link prediction could be tested here are the following: for students who consider becoming an entrepreneur, other common characteristics are: being Male, being between 18 and 25 years old, and having a degree in Data science Bachelor. Also among people who are between the ages 26 and 35, “paid job at an existing company” is a more common feature than for example “consider becoming an entrepreneur.”

A further advantage of the proposed approach is given by its explainability: The answer set itself describes the “solutions” for link prediction. By tracing back the applied rules used for inferring the answer set, specific choices can be illustrated for link prediction, i. e., which factors were responsible for establishing a specific link. In that way, ASP provides a transparent and interpretable approach for link prediction, integrating feature-rich networks complemented by background knowledge. In section 4.1, a hypothetical example showcasing link prediction enhanced with an attributive graph is given. That is, pairs of nodes in the interaction network are predicted to be linked, if they are similar in terms of their past behavior (captured by the existing number of common neighbors) or sharing attributes such as gender or area of study in the attributes network. This requires considering the topological information of both graphs, i. e., the list of nodes and edges, as well formalizing the rules defining common neighbors. Other rules then define link prediction based on the number of common neighbors in both graphs, as below.


 
  #**const** n=2.
  #**const** n_attrib=2.
  
  % a link is predicted when there are 2 common neighbors in the 
                interaction graph
  cn_lp(Y, Z) :- node(Y), node(Z), not edge(Y, Z), Y!=Z,  n=#count{X:c(X,
             Y, Z)}.
  % a link is predicted  when there are 2 common neighbors in the 
              attributive graph
  cn_lp(Y, Z) :- node(Y), node(Z), not edge(Y, Z), Y!=Z,  n_attrib=#count{
            X:c_attrib(X, Y, Z)}.
 


These rules simply state for a pair of distinct nodes *Y, Z*, which are not linked by an edge, a link is predicted between them when they have n (or n_attrib) common neighbors in the interactions or the attributive graph, respectively. Of course, the names can always be chosen to be more descriptive so that the logical statement resembles natural language more (link_predicted_based_on_common_neighbors instead of cn_lp). Basic understanding of logical expressions is enough to make sense of the rules. The answer set then itself captures the respective cn_lp facts, together with all those (new) facts that were applied in the solving process. Taken together, this then supplies an explanation as a trace of the applied rules, which can of course be complemented with further information such as, e. g., topological features in the form of statistical network descriptors.

## 6. Conclusions

In this paper, we proposed using ASP to incorporate background knowledge to the link prediction problem, which is not possible using some other approaches, for example, using standard social network analysis methods, e.g., proximity-based or path-based methods. In that way, we also introduced the application of ASP as a novel approach for link prediction. We explored that using a real-world data set capturing networks of face-to-face proximity at a student event: The dataset is relatively sparse, thus the link prediction problem is quite difficult, and becomes even more challenging in the context of the cold start problem. Therefore, the application of background knowledge proved to be especially relevant.

Our experiments using a standard common neighbors approach for link prediction showed, that providing background knowledge considerably improved the prediction performance. Furthermore, we showed how ASP can be conveniently applied in such a knowledge-based approach, in particular also relating to explanation-aware techniques since the result of ASP, i. e., the answer set, can be directly mapped to extensive explanations on the link prediction method. In this paper, we thus specifically demonstrated how to improve link prediction with contextual domain knowledge modeled using ASP – as a “proof of concept” of its applicability for link prediction. Furthermore, we demonstrated its advantages like explainability and enabling a simple formalization and refinement of domain knowledge.

For future work, we aim to extend and refine the model further, investigating different theory-based formalizations, like structural holes and social capital (Burt, 2002), and social roles (Scripps et al., 2007). Further future directions include the characterization of unpredicted links and extending the features used for the prediction toward temporal relationships, the order of the interactions, and information coming from the duration of conversations, as well as the existence of multiple edges–toward advanced link prediction in feature-rich complex interaction networks (Interdonato et al., 2019).

## Data Availability

The datasets generated for this study are available on request to the corresponding author.

## Author Contributions

ÇG and MA conceived of the idea, interpretation of the data and wrote the manuscript. ÇG implemented the method and ran the experiments.

### Conflict of Interest Statement

The authors declare that the research was conducted in the absence of any commercial or financial relationships that could be construed as a potential conflict of interest.

## Footnotes

^1^Available at: https://potassco.org/

^2^Participants were invited to wear RFID proximity tags. Study participants also gave their written informed consent for the use of their data in scientific studies. Data were collected in an anonymous way.

^3^http://www.sociopatterns.org/

^4^https://github.com/rafguns/linkpred/
